# Social Vulnerability and Surgery Outcomes: A Cross-sectional Analysis

**DOI:** 10.21203/rs.3.rs-3580911/v1

**Published:** 2023-11-28

**Authors:** Mohamed Abdelhack, Sandhya Tripathi, Yixin Chen, Michael S. Avidan, Christopher R. King

**Affiliations:** Department of Anesthesiology, Washington University School of Medicine, St. Louis MO; Krembil Centre for Neuroinformatics, Centre for Addiction and Mental Health, Toronto, ON; Department of Anesthesiology, Washington University School of Medicine, St. Louis MO; Department of Computer Science, Washington University in St. Louis, St. Louis MO; Department of Anesthesiology, Washington University School of Medicine, St. Louis MO; Department of Anesthesiology, Washington University School of Medicine, St. Louis MO

**Keywords:** Social vulnerability, postsurgical complications, housing status, socioeconomic status, sex interaction

## Abstract

**Background:**

Post-operative complications present a challenge to the healthcare system due to the high unpredictability of their incidence. However, the socioeconomic factors that relate to postoperative complications are still unclear as they can be heterogeneous based on communities, types of surgical services, and sex and gender.

**Methods:**

In this study, we conducted a large population cross-sectional analysis of social vulnerability and the odds of various post-surgical complications. We built statistical logistic regression models of postsurgical complications with social vulnerability index as the independent variable along with sex interaction.

**Results:**

We found that social vulnerability was associated with abnormal heart rhythm with socioeconomic status and housing status being the main association factors. We also found associations of the interaction of social vulnerability and female sex with an increase in odds of heart attack and surgical wound infection.

**Conclusions:**

Our results indicate that social vulnerability measures such as socioeconomic status and housing conditions could be related to health outcomes. This suggests that the domain of preventive medicine should place social vulnerability as a priority to achieve its goals.

## Background

Post-operative complications are adverse events that could occur after a surgical procedure. They occur in about a third of surgeries with the majority of these complications being minor (1). Multiple factors impact post-operative complications. Patient and hospital-level factors have been well studied, but understanding the social determinants of health (SDOH) for patient outcomes has become an important area of research. A key difficulty in research on SDOH is that electronic health records (EHR) and administrative data rarely contains accurate measures of these factors. Racial disparities in surgical outcomes and complications have been investigated directly, as race is one of the few SDOH routinely recorded in administrative data (2). A popular approach relies on the legacy of segregation in residential housing; in many areas of the United States economic status, racial, ethnic, and language groups are relatively concentrated in space. One attempt to unify economic, personal, social, and ethnic disadvantage into a single metric is the CDC’s Social Vulnerability Index (SVI) (3). Originally, the SVI was intended to assess the resilience of a community to disasters, such as environmental catastrophes.The SVI is freely available at the census tract level and defined using 16 variables extracted from US Census and survey data. These variables are organized into four “themes”: Socioeconomic status (poverty related variables), Household characteristics (age, disability, and English language proficiency), Racial and ethnic minority status, and Housing type & transportation.

The relationship between SVI and health outcomes has been explored in several existing works.

Prior to the formal definition of SVI, the relationship between socioeconomic status (SES) and the health outcomes was studied by using median household income of the residential zip code. A previous study observed that out of the patients admitted to hospital for acute pulmonary embolism, patients from lower median-income neighborhoods had a higher incidence of mortality compared to patients residing in higher SES zip codes (4). In addition to studying the above relationship, Sparrow et al. (5) also explored the impact of race/ethnicity on in-hospital complications in patients undergoing left atrial appendage closure (LAAC) and found higher incidence among Black patients in comparison to White patients; no significant impact of household income as a proxy of SES. CDC’s SVI has been correlated to worse surgical outcomes in cancer surgeries (6) and in hepatopancreatic surgeries (7), post-surgical complications in colon resection (8), colectomy (9), and esophagectomy (10). Most of the previous studies focused on one surgery type at a time or one adverse outcome making the results not very generalizable and increasing the risk of publication bias. In addition, the populations these studies used are often highly imbalanced focusing on high or low SVI groups.

Moreover, sex and gender have been known to also have a contribution to experiences and outcomes of health (11–13). Females were found to have more difficulty in recovering from environmental hazards (14–21). Previous work has studied the postsurgical complications in females and women and their relation to SVI but they fell short of comparing the additional risk that is associated with sex and gender (22).

Our objective is to determine the association between SVI and multiple post-surgical complications across a wide range of surgical procedures and its interaction with biological sex. By using a sample of over 50,000 patients and with physician confirmed comorbidities, we conduct a cross-sectional analysis using the records of a quaternary academic medical center drawing from the St. Louis bistate area. This region is characterized by large disparities in SVI (23), strong residential segregation in SDOH (24), and an almost equal representation of high and low SVI populations. Our data includes multiple surgery types to disentangle surgery- or disease-specific factors from general changes in risk. We find that higher SVI is associated with an elevated occurrence of only some complications, and that some complications have significant interaction with the patient’s biological sex. The associations were driven by both the socioeconomic status and housing conditions components of the SVI. Our results highlight the effects of inequality on the outcomes of surgery and the importance of investing resources to mitigate its effects in health care.

## Methods

### Patient Data Collection

Data was retrospectively extracted from the EHR and administrative records of Barnes Jewish Hospital between 2012 and 2018. The Human Research Protection Office at Washington University in St Louis, USA approved this study and granted a waiver of informed consent. The dataset contains patients’ demographics, billing address, preoperative laboratory measurements, medical and surgical history, physical examination, surgery details, and postoperative outcomes. It is described in detail in (25, 26) and was used in several previous studies (27–32). We included only addresses in Missouri and Illinois, as the density of patients outside these states was too low.

We retrieved SVI values from the Center for Disease Control (CDC) released for 2018. We mapped patient addresses to census tracts using ArcGIS Pro with the Business Analyst toolbox and aggregated outcome rates by census tract for visualization. We mapped the SVI data by census tract to each patient.

We selected a group of postoperative outcomes to analyze: 30-day mortality, congestive heart failure, deep venous thrombosis, leg blood clot, heart attack, surgical wound infection, pneumonia, nerve injury, abnormal heart rhythm, acute kidney injury, and delirium. All outcomes are incident, and they are defined in prior work (25).

### Statistical Analyses

We constructed two levels of modeling to account for different covariate factors in each level. We fit logistic regression models of the postoperative outcomes with the overall summary percentile ranking of SVI (RPL_THEMES) as an independent variable controlling for age, sex, race, and surgical specialty for level 0 and additionally for body mass index (BMI), smoking status, and diabetes for level 1 model. From these model coefficients, the odds ratio is reported between the two ends of ranking of the SVI values. A model with a SVI:sex interaction term was also computed. For the main and sex interaction effects, we accounted for multiple comparisons using the Benjamini-Hochberg false discovery rate correction (33). For the fine grained thematic effect models, we did not apply multiple comparison correction as associations were already confirmed from the main effects models.

## Results

**Figure 1:**
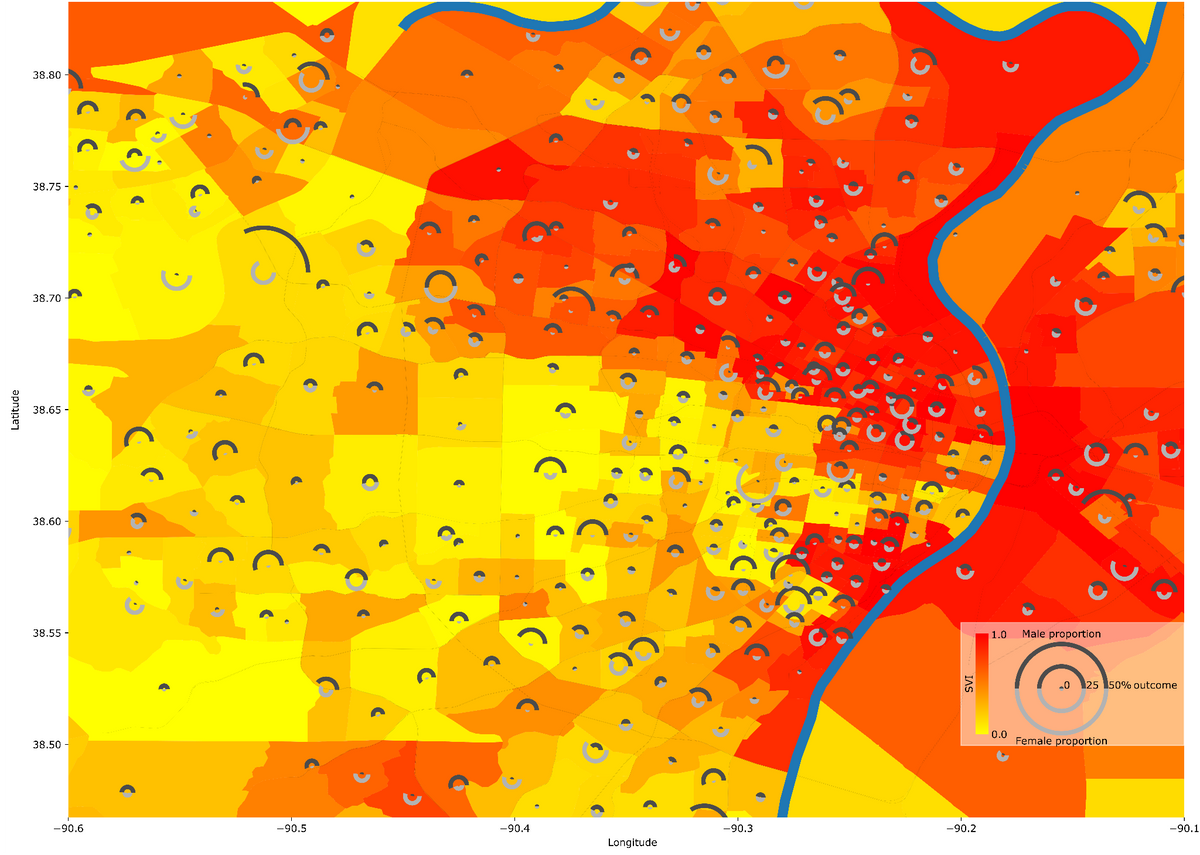
Sample outcome mapped with social vulnerability index (SVI): Mapping of SVI in counties of St. Louis bistate area with the odds of a sample outcome (abnormal heart rhythm) stratified by sex-at-birth for each census tract. Background color specifies the SVI value for the census tract and the pie chart radius indicated the proportion of the census tract population with the adverse outcome while the portion of the pie indicates the sex percentages. Counties with less than 20 subjects are omitted.

**Figure 2:**
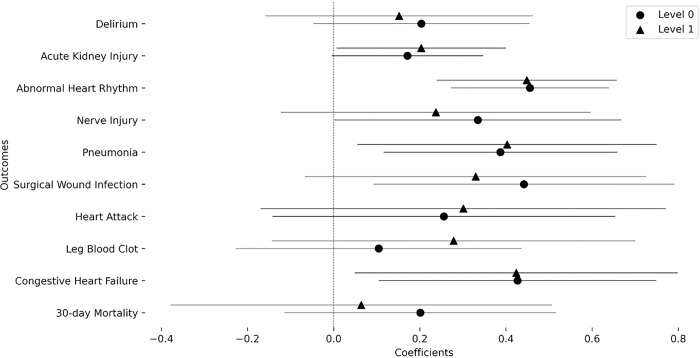
Coefficients of the overall SVI value in the association models for each outcome: Models of level 0 and level one are also compared to indicate the mediation effects.

Table 1 contains descriptive statistics of the included population. Female participants had a higher SVI albeit with a small difference in medians (Median SVI: Male = 0.48, Female = 0.49). Figure 1 shows a map of the St. Louis bistate area overlaid with a representative result of the abnormal heart rhythm outcome rate stratified per census tract and sex with sex percentages indicated. It also depicts the social vulnerability index (SVI) that shows a high discrepancy in SVI where the areas in the north and around the Mississippi river show relatively high SVI in comparison to the south western regions. The transition between low and high SVIs is abrupt and indicates the high levels of inequality within the community. It also visually depicts the increase in abnormal heart rhythm frequency in areas of high social vulnerability. This is further depicted in Fig. 2 where the coefficient of the SVI is 0.456 (*p*_FDR_=1.14×10^−5^) indicating that a transition from an SVI of 0 to 1 is associated with an increase in the odds of abnormal heart rhythm by 1.577 times. In addition to observing the above mentioned significant association at the level 0 modeling variables containing age, sex, race, surgery speciality, this significant relation was also observed in level 1 of modeling that includes smoking, diabetes status, and body mass index (coefficient = 0.448, *p*_FDR_=2.54×10^−4^, odds increase = 1.566). However, for congestive heart failure (coefficient = 0.427, *p*_FDR_=0.031, odds increase = 1.532), surgical wound infection (coefficient = 0.442, *p*_FDR_=0.033, odds increase = 1.555), and pneumonia (coefficient = 0.**387**, *p*_FDR_=0.**026**, odds increase = 1.**473**) the significant association of increasing outcome odds with an increase in SVI was only observed at level zero modeling but not at level one. The remaining outcomes did not show significant associations at any level. Complete statistical results are shown in Table 2.

**Figure 3:**
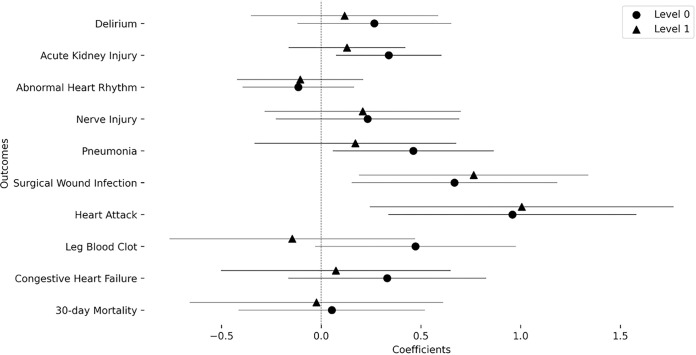
Coefficients of the interaction of overall SVI value with sex in the association models for each outcome: Models of level 0 and level one are also compared to indicate the mediation effects.

In order to investigate the effect on different sexes-at-birth, we added a sex interaction term (Fig. 3). It showed that SVI associations were further increased by sex being female for the surgical wound infection (coefficient = 0.668, *p*_FDR_=0.041, odds increase = 1.950) and heart attack (coefficient = 0.959, *p*_FDR_=0.025, odds increase = 2.610) outcomes. This was robust for level 1 as well in both outcomes (Surgical wound infection: coefficient = 0.764, *p* = 0.048, odds increase = 2.148; Heart attack: coefficient = 1.005, *p*_FDR_=0.048, odds increase = 2.731). The remaining outcomes did not show any significant sex interaction. Complete statistical results are shown in Table 3.

**Figure 4:**
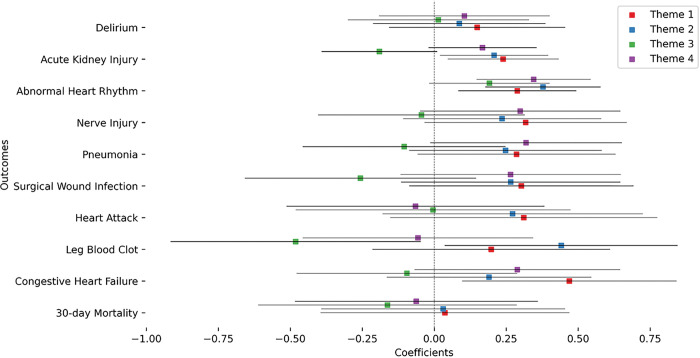
Coefficients of the thematic SVI value in the association models for each outcome: Models of level 1 only are displayed here.

**Figure 5:**
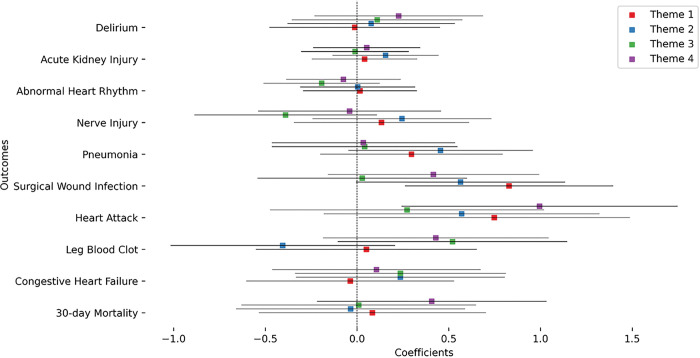
Coefficients of the interaction of thematic SVI value with sex in the association models for each outcome: Models of level 1 only are displayed here.

We then performed a more fine grained analysis of the theme-based social vulnerability index to pinpoint the form of vulnerability that contributes the most to the increase in odds of post-surgical outcomes. We first investigated the correlations between the different themes and their correlation to the overall SVI (Figure S1). Theme 1 had the highest correlation with the overall SVI while theme 3 had the lowest. Theme 2 and 3 had a negative correlation while themes 1 and 2 had the highest correlation. Theme 1 (Socioeconomic status) and theme 2 (Household composition) had the most abundant significant main associations with three outcomes each (Fig. 4). Theme 3 (Minority status and language) and theme 4 (English Language Proficiency, housing, and transportation) had one significant association each. Oddly, minority status and language had a negative coefficient meaning it contributed to decrease the odds of leg blood clot outcome (coefficient=−0.481, *p* = 0.030, odds = 0.617). Complete statistical results are shown in **Table S1**. Sex interaction analysis (Fig. 5) showed significant associations for theme 1 for both surgical wound infection and heart attack. Theme 4 had a significant interaction term association only for the heart attack outcome. In both the cases, the significant interactions indicated an increase in odds for females. Complete statistical results are shown in **Table S2**.

## Discussion

In this study, we investigated the association of tract-wide social vulnerability with the outcomes of surgical operations. We found abnormal heart rhythm, a cardiovascular complication, to be associated with the overall vulnerability. Socioeconomic status and housing status were some of the vulnerabilities that contributed to the overall associations with the outcome complications.

Our findings are in line with previous literature connecting social vulnerability with health outcomes where Hyer et al. (6) showed that cancer patients from high-SVI counties more frequently had a postoperative complication and had an extended length of stay. Further studies also showed the association between SVI and the overall group of post-operative complications that includes pulmonary failure, pneumonia, myocardial infarction, deep venous thrombosis, pulmonary embolism, renal failure, surgical site infection, gastrointestinal bleeding, and postoperative hemorrhage (7, 8). Stuart et al. (10) studied individual postoperative complications and found that esophagectomy patients with high SVI had greater rates of pneumonia, jejunal feeding-tube complications, and unplanned readmission. Carmichael et al. (9) also studied specific postoperative complications and found that colorectal surgery patients in the highest SVI quartile have increased risk of mechanical ventilation for more than 48 h postoperatively, surgical site infection, and sepsis/septic shock.

We also found other complications such as congestive heart failure, surgical wound infection, and pneumonia that have significant SVI association only at the level zero modeling. This suggests that in the tracts with high social vulnerability, there is a high incidence of smoking, diabetes, and unhealthy BMI. This challenges the findings of (34, 35) that found that smoking was not associcated with medical complications (myocardial infarction, pneumonia, etc) but is associated with surgical complications in arthroplasty patients. However, postoperative delirium was shown to be higher in patients with smoking history (36). Covarrubias et al. (37) observed that the likelihood of post-operative pulmonary complications increased with increasing body mass index in patients undergoing trauma laparotomy. Zhang et al. (38) found that lower body mass index and the presence of diabetes is a risk factor for postoperative pneumonia in craniotomy patients. A systematic review (39) noted that obese patients had significantly higher odds of post-operative atrial fibrillation when compared with non-obese patients undergoing cardiac surgery. These all point that the effect of SVI can be observed through the general health of the patients entering surgery due to smoking and food choices.

By investigating the sex interaction terms, we found that heart attack and surgical wound infection were specifically associated with females. Previous study noted the increased occurrence of post-surgical complications in females. Sah et al. (40) noted that females have a higher incidence of postoperative complications (as defined by POSSUM) after gastric cancer surgery and found patient gender to be a risk factor for post-operative complications. Aghdassi et al. (41) investigated gender as a risk factor for surgical site infections (SSI) and found that even though the incidence rate ratio and the adjusted odds ratio for SSI were significantly higher for male patients, for heart and vascular surgery, SSI-rates were significantly higher for female patients. Kim et al. (42) identified that female sex is one of the risk factors of postoperative hematoma after biportal endoscopic spinal surgery.

We also found that out of the sub-indices for vulnerability, socioeconomic status (SES) and household composition to be the most influential. Those two factors, in our study, are used as a proxy for the individual vulnerability of each patient. This is in contrast with the other two themes (household composition & disability and minority status & language) where they represent the community effect on the patient since we already include age and race as covariates in our model. These covariates are sub-measures of the two themes and they are measured at the individual level. An attempt to separate these two effects is made by Bonner et al. (43) where they studied the association between the racial disparitites in outcomes and the corresponding healthcare cost. Our findings line up with previous studies that found that liver transplants patients with medcaid or medicare (a proxy for SES) had higher postoperative mortality compared to the patients with private insurance (44). A systematic review found that socioeconomic status and patient demographics both impact the patient-reported outcomes post orthopaedic surgery (45). Measurements of SES by median neighbourhood household income (categorized into quintiles) revealed that lower SES is associated with fewer days alive and out of hospital post major elective non cardiac surgery (46). Ambur et al. (47) used the NIS database of more than 2 million patients undergoing cholecystectomy and found that SES had a negative impact on postoperative outcomes, in addition to being on Medicaid increasing portoperative mortality. Mheaffey et al. (48) investigated a sample of 44451 patients where distressed communities index (DCI) was used as a proxy for SES in the clinical quality improvement data and risk models. They found that higher DCI was associated with increased rate of postoperative complications and resource utilization even after ACS-CSQIO risk adjustment further demonstrating surgical outcome disparity based on community level socioeconomic factors.

The effect of socioeconomic status as reflected by belonging to a certain income region and housing was particularly affecting females’ incidence of heart attack and surgical wound infection. Previous studies investigated the intersectional effect of gender and SES on internalizing symptoms and found no interactive negative effects for female gender and low SES in a sample of predominantly lower-SES adolescents (49). However, they did observe that females at the lowest levels of caretaker education and household income (indices of SES) were at higher risk. In another study on female adolescents, Madhushanthi et al. (50) found that low SES impacted the executive function test score significantly in Sri Lanka. Leng et al. (51) showed that increased risk of hypertension in the lowest categories of all SES indicators was most evident in women, whereas men revealed less consistent associations. Hinz et al. (52) showed that individually being a female and belonging to low SES strata was associated with poor sleep quality. All those indicators of health could contribute to the disproportionate effect that females are exposed to from social vulnerability.

Our investigation here is limited by multiple factors. First of all, SVI as a proxy will tend to understate true associations vs. measuring the patient level factors. Additionally, our study population introduces biases where the population that is able to receive a surgery is the insured or the more financially stable within their regions. Also, the regional nature of the study means there are possibly region-specific variables that could bias our results. However, we argue that location specificity is actually an advantage as it provides a balanced representation of different SVIs from one region. Our results only include a limited number of covariates, while it is possible for other terms not included in our model could explain more variance. Finally, the use of uncorrected statistics for and theme-based analysis could lead to more type-2 errors but we opted to not correct them since the relations were already established in the overall SVI models. Regardless of the biases and limitations, the results are useful for planning interventions to target preoperative optimization and postoperative support services.

## Conclusions

Our results indicate that social vulnerability could be related to adverse outcomes of surgery and that females are more affected. The socioeconomic status and housing were the most significantly associated factors which indicates the importance of prioritizing welfare as a method of improving surgical outcomes. These results could serve as a guidance for the public health policy field as social vulnerability could be viewed as adding an accruing cost to the healthcare system. We can also argue through these results that decreasing social vulnerability is an important step towards achieving prevention-based healthcare.

## Figures and Tables

**Table 1:** Participant cohort statistical characteristics

Characteristic	Total (n = 57811)	Male (n = 27980)	Female (n = 29831)
Age, Median (IQR)	57 (23)	58 (22)	55 (24)
Sex, n (%)	57819	27980 (48.40%)	29831 (51.60%)
BMI, Median (IQR)	28.65 (9.3)	28.32 (7.9)	29.04 (10.8)
Smoking Status Yes N (%)	27263 (52.38%)	14742 (59.13%)	12521 (46.16%)
Diabetes N (%)	4139 (7.95%)	2120 (8.50%)	2019 (7.44%)
Surgery Type N (%)			
Orthopaedic	14521 (25.1)	7087 (25.3)	7434 (24.9)
Gynecology	6495 (11.2)	7 (0.03)	6488 (21.7)
Cardiothoracic	6227 (10.8)	1053 (12.9)	2614 (8.8)
Urology	5903 (10.2)	4062 (14.5)	1841 (6.2)
Neurosurgery	5250 (9.1)	2773 (9.9)	2477 (8.3)
Otolaryngology	4236 (7.3)	2441 (8.7)	1795 (6.0)
General Surgery	3643 (6.3)	1983 (7.1)	1660 (5.6)
Vascular	3145 (5.4)	1677 (6.0)	1468 (4.9)
Colorectal	1994 (3.4)	1053 (3.8)	941 (3.2)
Transplant	1767 (3.1)	960 (3.4)	807 (2.7)
Hepatobiliary	1411 (2.4)	653 (2.3)	758 (2.5)
Minimally Invasive Surgery	925 (1.6)	366 (1.3)	559 (1.9)
Plastic	889 (1.5)	579 (2.1)	310 (1.0)
Unknown	805 (1.4)	369 (1.3)	436 (1.5)
Others	600 (1.0)	357 (1.3)	243 (0.8)
Race/Ethnicity			
White	43177 (74.7)	21302 (76.1)	21875 (73.3)
Black	11418 (19.8)	4963 (17.7)	6455 (21.6)
Asian	417 (0.7)	171 (0.6)	246 (0.8)
Other	55 (0.1)	22 (0.1)	33 (0.1)
Unknown	2744 (4.7)	1522 (5.4)	1222 (4.1)
SVI-total, Median (IQR)	0.48 (0.49)	0.48 (0.49)	0.49 (0.50)
SVI-Socioeconomic Status, Median (IQR)	0.49 (0.51)	0.47 (0.50)	0.50 (0.51)
SVI-Household Characteristics, Median (IQR)	0.55 (0.50)	0.54 (0.50)	0.55 (0.50)
SVI-Racial and Ethnic Minority Status, Median (IQR)	0.40 (0.47)	0.38 (0.48)	0.41 (0.48)
SVI-Housing Type/Transportation, Median (IQR)	0.52 (0.48)	0.51 (0.49)	0.53 (0.48)
Outcomes N (%)			
30-day Mortality	514 (0.978)	280 (1.123)	229 (0.844)
Congestive Heart Failure	709 (1.348)	431 (1.729)	269 (0.992)
Leg Blood Clot	536 (1.019)	305 (1.223)	224 (0.826)
Heart Attack	396 (0.753)	247 (0.991)	140 (0.516)
Surgical Wound Infection	623 (1.185)	339 (1.36)	273 (1.007)
Pneumonia	830 (1.578)	460 (1.845)	365 (1.346)
Nerve Injury	827 (1.573)	423 (1.697)	393 (1.449)
Abnormal Heart Rhythm	2483 (4.722)	1501 (6.021)	955 (3.521)
Acute Kidney Injury	2650 (5.242)	1567 (6.565)	1066 (4.054)
Delirium	1695 (40.992)	967 (39.909)	716 (42.267)

**Table 2:** Association of SVI with postoperative outcomes. It shows odds ratio and p-values. Bolded rows are statistically significant after false discovery rate correction.

Outcome Variable	Level	Odds ratio (CI)	*p*-value	*Adjusted p*-value
30-day Mortality	0	1.223 (1.370)	0.211	0.234
	1	1.066 (1.557)	0.777	0.777
Congestive Heart Failure	**0**	**1.532 (1.389)**	**9.45×10^−3^**	**0.031**
	1	1.528 (1.455)	0.027	0.089
Leg Blood Clot	0	1.110 (1.394)	0.537	0.537
	1	1.321 (1.525)	0.196	0.263
Heart Attack	0	1.292 (1.488)	0.207	0.234
	1	1.351 (1.601)	0.210	0.263
Surgical Wound Infection	**0**	**1.555 (1.418)**	**0.013**	**0.033**
	1	1.390 (1.486)	0.103	0.205
Pneumonia	**0**	**1.473 (1.312)**	**0.005**	**0.026**
	1	1.496 (1.415)	0.023	0.089
Nerve Injury	0	1.397 (1.395)	0.049	0.094
	1	1.267 (1.433)	0.196	0.263
**Abnormal Heart Rhythm**	**0**	**1.577 (1.201)**	**1.14×10^−6^**	**1.14×10^−5^**
	**1**	**1.566 (1.232)**	**2.54×10^−5^**	**2.54×10^−4^**
Acute Kidney Injury	0	1.187 (1.192)	0.056	0.094
	1	1.225 (1.217)	0.043	0.107
Delirium	0	1.226 (1.285)	0.112	0.160
	1	1.164 (1.364)	0.338	0.375

**Table 3:** Interaction term of SVI and female sex for post-surgical complications. Bolded rows are statistically significant after false discovery rate correction.

Outcome Variable	Level	Odds ratio (CI)	*p*-value	*Adjusted p*-value
30-day Mortality	0	1.054 (1.596)	0.824	0.824
	1	0.976 (1.889)	0.941	0.941
Congestive Heart Failure	0	1.392 (1.641)	0.191	0.273
	1	1.076 (1.779)	0.803	0.892
Leg Blood Clot	0	1.603 (1.654)	0.066	0.132
	1	0.864 (1.849)	0.644	0.805
**Heart Attack**	**0**	**2.610 (1.864)**	**0.003**	**0.025**
	**1**	**2.731 (2.139)**	**0.010**	**0.048**
**Surgical Wound Infection**	**0**	**1.950 (1.674)**	**0.011**	**0.041**
	**1**	**2.148 (1.777)**	**0.009**	**0.048**
Pneumonia	0	1.588 (1.497)	0.025	0.062
	1	1.187 (1.656)	0.507	0.805
Nerve Injury	0	1.262 (1.585)	0.322	0.402
	1	1.231 (1.636)	0.406	0.805
Abnormal Heart Rhythm	0	0.892 (1.323)	0.420	0.467
	1	0.901 (1.371)	0.513	0.805
Acute Kidney Injury	0	1.402 (1.303)	0.012	0.041
	1	1.138 (1.339)	0.387	0.805
Delirium	0	1.304 (1.470)	0.176	0.273
	1	1.125 (1.600)	0.624	0.805

## Data Availability

The datasets generated and/or analysed during the current study are not publicly available due to containing sensitive patients’ information but are available from the corresponding authors on reasonable request.
